# Impact of interprofessional service-learning on the effectiveness of knowledge transfer of antimicrobial resistance to Hong Kong elders: a quasi-experiment

**DOI:** 10.1186/s13756-021-01011-9

**Published:** 2021-10-12

**Authors:** Anna C. Y. Lo, Joyce T. S. Li, Janita P. C. Chau, Samuel Y. S. Wong, David S. C. Hui, Vivian W. Y. Lee

**Affiliations:** 1 School of Pharmacy, Faculty of Medicine, The Chinese University of Hong Kong, Shatin, N.T. Hong Kong; 2 Centre for Learning Enhancement And Research, The Chinese University of Hong Kong (CLEAR), Room 502 Hui Yeung Shing Building, Shatin, N.T. Hong Kong; 3 The Nethersole School of Nursing, Faculty of Medicine, The Chinese University of Hong Kong, Shatin, N.T. Hong Kong; 4 The Jockey Club School of Public Health and Primary Care, Faculty of Medicine, The Chinese University of Hong Kong, Shatin, N.T. Hong Kong; 5 Department of Medicine and Therapeutics, Faculty of Medicine, The Chinese University of Hong Kong, Shatin, N.T. Hong Kong

**Keywords:** Interprofessional education, Antimicrobial resistance, Knowledge transfer, Service-learning, Elderly

## Abstract

**Background:**

Community perception on antimicrobials plays a role in driving the development of antimicrobial resistance (AMR). The aim of the study was to evaluate the impact of interprofessional service-learning on the effectiveness of AMR knowledge transfer in Hong Kong elders aged 65 or above and students from university and secondary schools.

**Methods:**

A quasi-experimental pretest–posttest controlled study was carried out from July 2018 to March 2019 for elderly subjects and a pre- and post-test were conducted in students from May to August 2018. Elderly subjects were recruited from the university-based community outreach program. The community outreach team consisted of both university and secondary school students. Students were provided with training of geriatric care and AMR before they reached out to the community. The one-to-one intervention with the aid of video and verbal explanation to educate the elderly about the definition, causes, and consequences of AMR, and preventive measures against AMR was provided. Questionnaires on knowledge of antibiotics and AMR were used as tools to reflect on the effectiveness for both students and elderly subjects. The questionnaire was completed twice, before and 1 week after the intervention. Chi-square test, *t* tests and regression analysis were used to analyze the data.

**Results:**

A total of 93 Chinese elders, 61 of them in the intervention group and 32 in the control group participated in the study. The score obtained by the intervention group increased from 40.1 to 83.3% (*p* < 0.001) following intervention, while that of control group increased from 33.0 to 44.0% (*p* < 0.001). The increase attained in the intervention group was significantly greater than that of the control group (*p* < 0.001). A total of 95 secondary students and 88 university students have completed the pre-post questionnaires with 42.21% and 13% increment in AMR knowledge after the training (*p* < 0.001).

**Conclusion:**

The significant change in knowledge level showed effective AMR knowledge transfer to both elders and students. The study could be used as a reference when allocating resources to implement effective interprofessional service-learning for better community health education in elderly populations.

*Trial registration*: This study was approved by the Chinese University of Hong Kong Survey and Behavioural Research Ethics Committee in December 2018 (Ref no. SBRE-18-214).

## Background

Antimicrobial resistance (AMR) is a fast-growing global issue that needs to be addressed promptly as it threatens the health of humans. It refers to the condition where microorganisms evolve upon exposure to antimicrobial drugs, rendering the medications ineffective [1]. The problem of AMR is very serious as it affects everyone around the world. A surveillance report conducted by the World Health Organization (WHO) stated that in 5 out of 6 WHO regions, over 50% of bacterial infection cases in hospitals and the community have reported resistance of *Escherichia coli* and *Klebsiella pneumoniae* towards third generation cephalosporin, and resistance of *Staphylococcus aureus* towards methicillin [1]. In Hong Kong, over 20% of *Staphylococcus aureus* infection cases have been shown to be resistant to methicillin [2]. With increasing cases of antimicrobial resistant infections, pharmacotherapy may be unsuccessful in a few years if measures to promote stewardship initiatives are not prioritised [3]. The uncontrolled development of antimicrobial resistant infections could result in 700,000 deaths annually and estimated economic losses of 2.9 trillion USD by 2050 [4, 5].

Misuse and overuse of antimicrobials clinically, and the agricultural practice of using antimicrobials for non-infection causes in animal husbandry are some of the causes that have driven the rapid growth of AMR [6]. One of the causes that could be addressed in the community is the public’s use and perception of antimicrobials. Common misuse that occurs in the community includes incompletion of treatment course and purchase of non-prescription antimicrobials. These actions further augment the misuse and overuse of antimicrobials, resulting in the further spread of AMR [7]. Therefore, it is important to educate citizens in the community as a way to address AMR. Both university and senior secondary school students are important target groups for AMR knowledge transfer because they are mature enough to understand the AMR issue and can also serve as ambassadors to raise AMR awareness and AMR prevention strategies. As for the elderly, they were the group with the highest percentage of giving 'I do not know' as responses in an AMR survey where true or false responses were required [8]. The survey results showed that although Hong Kong people in general may have better knowledge of antibiotics compared to the world, there is still a portion of people with misconceptions about antibiotics. Moreover, the awareness for AMR is still low in either Hong Kong or worldwide. It should be noted that the elders have the lowest level of understanding in antibiotics and AMR among the different age groups in Hong Kong [8].

Antibiotics are also prevalently used in Hong Kong. Compared to Europe, the use of non-prescription antimicrobials is more prevalent in Asian countries, with 58% use of antimicrobials being non-prescription use [9]. Therefore, there would be generally less guidance on the use of antimicrobials which increases risks of developing AMR. The lack of separation between prescribing and dispensing also increases chances of misuse and overuse of antimicrobials especially in private clinics where prescriptions are not verified by pharmacists. A systematic review and meta-analysis analyzed 38 studies from 24 countries and reported a pooled proportion of 78% non-prescription antibiotics being supplied by community pharmacies following a patient request [10]. Asia ranked the second highest in terms of regional supply of non-prescription antibiotics (65%) [10]. Non-prescription antibiotics were commonly supplied to patients with symptoms of urinary tract infections (68%) and upper respiratory tract infections (67%) [10]. Moreover, Hong Kong is in close proximity to China, where antibiotics could be easily assessed without any prescription [11]. It has been shown that 95.8% of pharmacies offer non-prescription antibiotics for adult respiratory infections [11]. This increases possibility that Hong Kong citizens could obtain antibiotics without proper counselling and are more prone to inappropriate use of the medications. Another geographical risk factor would be that Hong Kong has a high population density, which would facilitate the spread of bacteria and viruses, and so resulting in higher risks of developing AMR [12]. The Hong Kong Centre for Health Protection (CHP) suggested that health education materials, including posters, leaflets and videos, are to be utilized in community for raising public awareness. Seminars and campaigns are also recommended as a means to promote AMR awareness [13]. Current studies focused more on use of mass media in raising awareness in the general public, the results of these studies were a bit hit and miss, one of the concerns would be the passive intervention with use of mass media only [14–16]. There were also school-based intervention, which gave more promising results in raising awareness and knowledge on antibiotic and antibiotic resistance [17, 18]. To date, there is no study to document the impact of those educational interventions to the students and in the community. One of the reviews has shown that a more interactive form of intervention would be more effective than employing mass media solely when it comes to improving knowledge about antibiotics, but more research is needed to investigate the impact of communication interventions [19]. It was also suggested that there is limited scientific evidence regarding cause-effect relationship between intervention and knowledge improvement [20]. Therefore, the current study aimed to investigate the effectiveness of interprofessional service-learning on the AMR knowledge transfer to elders and to secondary school and university students in Hong Kong.

## Methods

### Secondary school and university students

Our community outreach team, Community Health and Medication-safety Promotion Inter-school Outreach Network (CU CHAMPION) of the Chinese University of Hong Kong (CUHK) outreach services, is an interprofessional outreach team conducting service-learning programs for university students. Our students are from the faculty of medicine, faculty of social sciences and faculty of sciences at CUHK. In addition, we also recruited senior secondary school students (Form 4 or above) from 6 band 1 (top 1/3) English as Medium of Instruction (EMI) secondary schools located in 5 different districts in Hong Kong. Secondary schools in Hong Kong are classified into band 1 (top 1/3), band 2 (middle 1/3), and band 3 (bottom 1/3) based on their academic performance. Teachers from each secondary school could nominate up to 20 students who were interested in pursuing study in health-related undergraduate programs to join CU CHAMPION at CUHK. All university and secondary students underwent e-learning and training workshops on AMR in May and June before joining the community service in July and August 2018. Students were required to have 100% participation and attendance rate for both e-learning and training workshops. The current project was also introduced to both university and secondary school students so that they understood the objectives and the logistics of the study. Students had to interview elderly subjects and to conduct questionnaires.

### Elderly subjects

The study was conducted on Hong Kong elderly aged 65 or above. Participation inclusion criteria were Hong Kong elderly aged 65 or above, and could communicate in Cantonese. Exclusion criteria were memory impairment screening (MIS) score of 4 or below, or diagnosed with dementia. They were excluded since they are prone to giving extreme results and could mask the true effectiveness of the intervention [21, 22].

During 2018 July to August, around 1600 elders from 36 elderly centers were reached. They completed a questionnaire about their general health status with the assistance of student volunteers. The elderly centers were community centers located within public estates across 13 districts in Hong Kong. Subjects recruited through the outreach services were screened according to the inclusion and exclusion criteria by our CU CHAMPION students. After communicating with the elderly centers on the consent and accessibility, 10–12 subjects were invited from each of the centers that agreed to work on the project. The subjects were assigned a number and selected randomly with the use of an online randomizer, https://www.random.org/. The study aimed to achieve a 1:1 allocation between intervention and control group, however, due to practical restraints such as considerations of the elderly centers and accessibility to information, the allocation was stepped down to a 2:1 allocation between intervention and control group. Although the educational session was also provided to the control group after completing the posttest, some elderly centers insisted to only take part for the intervention group. Moreover, some elderly centers could not provide contact of participants prior to the intervention, which was needed to facilitate the conduction of the control group interview, as without the contact, two face-to-face sessions were needed and that could cause possible nuisance to elderly and extra time and resources. Therefore, allocation of participants to intervention and control group were decided with the above considerations. Informed consents were obtained from all subjects upon invitation to take part in the study.

### Materials and instrument

Questions adapted from WHO survey on antibiotics resistance were used to assess the understanding on AMR by the elders and students. Students were asked to complete the pretest questionnaire before they attended the e-learning and workshops. They were asked to complete the posttest questionnaire after the e-learning and workshops. The pretest questionnaire included two parts, five true or false questions on knowledge on the use of antibiotics and 6 true or false questions on knowledge about AMR. The posttest questionnaire included three parts, the first two parts were the same as the pretest questionnaire, i.e., a total of eleven true or false questions regarding knowledge on the use of antibiotics and about AMR. An additional part of feedback on intervention was included to evaluate different materials used in the intervention, where nine statements were given, and subjects expressed their stances by agreeing the statement from a scale of one to six. One score was given in accordance to each correct answer, the score out of a total of 11 was the tool to assess the level of understanding by the subject. The difference in the score of the pretest and posttest questionnaire was used to reflect the effectiveness of the knowledge transfer.

For the elders in the intervention group, pretests were conducted face-to-face right before the implementation of intervention, while posttests were conducted via phone 1 week after the education intervention. For the elders in the control group, pretests were conducted via phone, and posttests were conducted face-to-face individually. Face-to-face interview was adopted in the intervention group’s pretest and the control group’s posttest, so that the educational session could be provided subsequently. Phone interview was adopted in the other tests for convenience.

### Interventions

The study was conducted individually, where the elderly subjects met with one investigator in the session. The intervention session was timed to be within 15 min. The sessions were conducted in private meeting rooms in district community elderly centers. The sessions were carried out by final-year students and supervised by a clinical pharmacist from the School of Pharmacy, CUHK. Before assisting in the educational session, students were required to attend a 45-min lecture on AMR delivered by a registered pharmacist and read specific materials about AMR to be able to facilitate teaching in the study.

For the intervention group, an informative session was provided. The intervention content was standardized with a specific timetable and content, beginning with a 5-min video session, then followed by a 10-min face to face verbal health education done by the investigator. The exact contents would be discussed in the following section. The investigators were given guidelines on what educational points must be covered within the 10 min, they were also encouraged to invite the subject to talk back and strengthen the knowledge obtained and to give feedback to the investigators. For the control group, they received no intervention during the 1-week time between pretests and posttests. Educational session was given after completion of posttests to be ethically fair to the control group.

### Educational content

Efforts should be made on raising awareness on AMR and promoting appropriate use of antimicrobials [13]. Additional emphasis should be placed on correcting existing misconceptions regarding the use of antimicrobials [13]. In accordance to the advice, the content that were covered in the study includes: definition, causes and consequences of AMR, and preventive measures against AMR. It is important to mention the cause and consequences because when people understand that they are at risk of the condition and the subsequent serious consequences, they would be more willing to take action to tackle the specific health issue [23]. With the incomplete understanding of AMR that the public do not think they are related to the issue, it was also crucial for them to be aware of their ability to make a difference in deterring AMR, which could provide incentives for them to take initiatives [24, 25].

The 5-min video introduced definition, causes and consequences of AMR, and preventive measures against AMR For the choice of using a video, it could deliver basic concepts within a short period of time. It was found that the use of video in health promotion can bring a positive change in the knowledge of the public [26]. Videos are often easy to watch and listen, and therefore could be effective in raising understanding [19]. In addition, elderly patients usually have poor eyesight. It was difficult for them to read educational pamphlets with small print. The government estimated that 25–30% of elders in Hong Kong could not read [27]. Therefore, a video was used in the study to provide a general overview of the issues of AMR.

However, the insufficiency of using solely video in education is that it lacks an interactive component that could address individual’s needs, and so a face-to-face communication session was arranged to promote the major concepts. The verbal education content focused on preventive measures against AMR, as their actions is what matters most to prevent AMR. The interaction allowed two-way communications, where the needs and views of the subject could be known in the process and addressed immediately, which could increase the educational outcome that the gained knowledge would affect the practice [28]. It is particularly important in elderly education, since they are heterogeneous in nature and could have very different education bases due to differences in lifelong accumulation of cultural capital [29].

### Statistical analysis

The primary endpoint was the knowledge enhancement based on the scores between pretest and posttest for elders. Secondary endpoints included knowledge scores changes for students. The study had a level of significance of 0.05 and a power of 0.8. Estimated effect size according to previous similar studies conducting health education intervention was 0.5 [14, 30]. The study aimed to recruit a total of 144 subjects for the study to be statistically significant, with 96 subjects in the intervention arm and 48 subjects in the control arm. Descriptive statistics were used to analyze the baseline characteristics of the subjects. The study used a *p* value < 0.05 and a confidence interval of 95% for all analyses. 2-sided paired *t* tests were used to determine if there was a significant change between pretests and posttests scores. Independent *t* tests were used to compare changes in knowledge level between the intervention group and the control group. Regression was used to determine any differences between changes in knowledge level in subjects with different characteristics.

## Results

A total of 13 elderly centers worked with the project within the period from July 2018 to March 2019. The study invited 138 elderly subjects, and with a participation rate of around 70%, a total of 97 participants took part in the study. Four participants did not complete the study. Therefore, the study successfully interviewed a total of 93 participants, with 61 participants in the intervention group and 32 participants in the control group. The number of participants did not reach the proposed amount due to practical constraints such as availability of elderly centers and limited time. The demographic data was summarized in Table 1 with no significant differences between the intervention group and the control group except the living condition and the availability of carers. For the intervention group, there were significant differences in the pretest and posttest scores in both knowledge regarding antibiotics and AMR. It is shown that the rise in knowledge level on AMR is greater than that of antibiotics (Table 2). The participants scored over 90% in antibiotics knowledge, and although they performed poorer on AMR, they still achieved a score of 76%. These suggested the positive impact of the education intervention. Most questions were accurately answered except question 1. Majority answered question 1 wrongly at the pretest, and it was also the question with the lowest percentage of correct answers at posttest. Other questions on antibiotics at posttests had attained a correct answer rate of over 90%. Regarding questions on AMR, less than 50% obtained the correct answer for all six questions before intervention. After intervention, over 80% of participants answered correctly for question 7, 8, 9, 11. Only around 50% of participants correctly identified the false statement about definition of AMR. For question 10, only around 50% of participants correctly answered it at posttest.

**Table 1:** Demographic data of elderly subjects

	Intervention (n = 61)		Control (n = 32)		*p* value
	Mean (SD)	N (%)	Mean (SD)	N (%)	
Age	75.8 (6.51)		76.5 (6.31)		0.594
Sex					0.200
Male		12 (19.7)		3 (9.04)	
Education level					0.997
Never received education		20 (32.8)		10 (31.3)	
Primary education		25 (41.0)		13 (40.6)	
Secondary education		14 (23.0)		8 (25.0)	
Tertiary education		2 (3.28)		1 (3.13)	
Use of chronic medications					0.915
Yes		49 (80.3)		26 (0.813)	
Living condition					0.033*
Alone		13 (21.3)		15 (46.9)	
Accompanied by family member/maid		47 (77.0)		17 (53.1)	
Elderly homes		1 (1.64)		0 (0)	
Caregiver					0.035*
Self		41 (67.2)		30 (93.8)	
Spouse		13 (21.3)		1 (3.13)	
Children		4 (6.99)		1 (3.13)	
Maid		2 (3.64)		0 (0)	
Baseline knowledge					
Pretest score on antibiotics knowledge	61.6% 3.08 (1.55)		53.2% 2.66 (1.73)		0.249
Pretest score on AMR knowledge	20.8% 1.25 (1.48)		16.2% 0.97 (1.45)		0.387
Pretest total score	40.1% 4.41 (2.57)		33.0% 3.63 (2.67)		0.178

*p value < 0.05

**Table 2:** Knowledge of antibiotics and AMR in intervention group of elders (by question)

		Pretest	Posttest	*p* value
Knowledge on antibiotics				
1	Antibiotics can be used to treat cold and flu. (ANS: false)			< 0.001
	Correct	20 (32.8)	47 (77.0)	
	Incorrect/don’t know	41 (67.2)	14 (23.0)	
2	Antibiotics are used to target bacteria. (ANS: true)	< 0.001		
	Correct	36 (59.0)	58 (95.1)	
	Incorrect/don’t know	25 (41.0)	3 (4.90)	
3	I can buy the same antibiotics, or request them from doctors, if I am sick and they had helped me get better when I had the same symptoms before. (ANS: false)		0.0024	
	Correct	47 (77.0)	59 (96.7)	
	Incorrect/don’t know	14 (23.0)	2 (13.3)	
4	I can take antibiotics from friends or family, as long as they are used to treat the same illness (ANS: false)		< 0.001	
	Correct	43 (70.5)	56 (91.8)	
	Incorrect/don’t know	18 (29.5)	5 (8.2)	
5	It is fine to stop taking antibiotics once you start to feel better. (ANS: false)		< 0.001	
	Correct	42 (68.9)	59 (96.7)	
	Incorrect/don’t know	19 (31.1)	2 (13.3)	
Score on antibiotics knowledge	61.6% 3.08(1.55)	91.0% 4.55(0.85)	< 0.001	

The same set of questions were distributed to the student volunteers. A total of 146 students completed the survey (Table 3). Significantly higher number of students answered question 2 (from 84.9 to 93.2%, *p* = 0.036), question 3 (from 66.4 to 80.1%, *p* = 0.005), question 7 (from 84.2 to 94.5%, *p* = 0.003), question 8 (from 47.9 to 80.1%, *p* < 0.001), question 9 (from 84.2 to 93.2%, *p* = 0.019), and question 10 (from 67.8 to 84.2%, *p* < 0.001) correctly at the post-test as compared to the pre-test. Students’ score in antibiotics knowledge improved from 86.6 to 91.8% (*p* = 0.001), and their score in AMR improved from 74.9 to 89.6% (*p* < 0.001).

**Table 3:** Knowledge of antibiotics and AMR in students (by question correction rate)

Knowledge on AMR				
6	Antibiotic resistance occurs when your body becomes resistant to antibiotics, and they no longer work as well. (ANS: false)			< 0.001
	Correct	1 (1.64)	33 (54.1)	
	Incorrect/don’t know	60 (98.4)	28 (45.9)	
7	Bacteria resistant to antibiotics can be spread from person to person. (ANS: true)			< 0.001
	Correct	10 (16.4)	49 (80.3)	
	Incorrect/don’t know	51 (83.6)	12 (19.7)	
8	Inappropriate use of antimicrobials would accelerate the development of resistant microorganism, resulting in AMR. (ANS: true)			0.007
	Correct	21 (34.4)	58 (95.1)	
	Incorrect/don’t know	40 (65.6)	3 (4.90)	
9	Antibiotic-resistant infections could make medical procedures like surgery, organ transplants and cancer treatment much more dangerous. (ANS: true)			< 0.001
	Correct	17 (27.9)	53 (86.9)	
	Incorrect/don’t know	44 (72.1)	8 (13.1)	
10	I am not at risk of getting antibiotic-resistant infection, as long as I take my antibiotics correctly. (ANS: false)			< 0.001
	Correct	4 (6.56)	32 (52.5)	
	Incorrect/don’t know	57 (93.4)	29 (47.5)	
11	Maintaining good hand hygiene can help address the problem of AMR. (ANS: true)			< 0.001
	Correct	23 (37.7)	55 (90.2)	
	Incorrect/don’t know	38 (62.3)	6 (9.8)	
	Score on AMR knowledge	20.8% 1.25(1.48)	76.5% 4.59(1.33)	< 0.001
	Total score	40.1% 4.41 (2.57)	83.3% 9.16 (1.92)	< 0.001

## Discussion

The current study was similar to the survey conducted by CHP, stating that 40% of the elderly mistakenly believed that antibiotics can be used to treat cold and flu [8]. Sixty percent of respondent in the WHO survey also wrongly agreed with the statement [7]. It was also the question with the lowest percentage of correct answers at posttest. The observation was consistent with previous studies that suggested difficulty in educating the public on differences between bacterial and viral infection [31]. Therefore, the public failed to recognize the fact that cold and flu are viral infection and do not require antibiotics as treatment. Other questions on antibiotics at posttests had attained a correct answer rate of over 90%, suggesting that the information was easy to be introduced and obtained with simple tools and in limited time.

Regarding questions on AMR, less than 40% obtained the correct answer for each question at pretest before intervention. The observation is as predicted from the CHP and WHO survey, where more than half of the respondents have not heard of the term—AMR before [7, 8]. For question 6, only 1 person (1.64%) correctly answered. The statement about the definition of AMR was also hardly answered correctly in survey done by the CHP or WHO, with only 10% of respondents correctly identified that AMR do not refer to development of resistant of human body, but resistant of microorganisms. It suggested a low awareness of AMR among the public. After intervention, over 80% of participants answered correctly for question 7, 8, 9, 11, but for question 6, the rate of answering correctly was only 50%. The rates suggested that the concept of AMR is not known by much, and it is more difficult to introduce the concept in a short period of time to an elderly [7, 8]. As for or question 10, there were also only around 50% of participants correctly answered it at posttest. The observation suggested that the causes of AMR was not well understood by the public.

There were areas that requires more attention. It was noted that it is difficult to educate the public regarding differences between bacterial and viral infections, and corresponding treatment for them [18, 31]. Global and local surveys have also reported a lack of awareness regarding AMR [32]. The findings from the study corresponded to the previous data, as the rates of answering statements on these topics are low, and that remained lower than other topics after intervention. It is recommended that future education could place greater focus in educating different infections and definition and causes of AMR. For difficult topics, repeated education over longer duration is suggested to produce sustained effects [31].

Overall, from results from pretests, the observations were comparable and similar to surveys conducted by the CHP in Hong Kong and WHO globally, where respondents performed fairly or poorly in similar areas. It was noted that although Hong Kong elderly had a comparatively weaker knowledge base than other age groups in Hong Kong, their knowledge level were slightly better than the general level globally, which was observed from comparing the statistics from CHP and WHO. Also, the majority of participants of the study had low education level; yet, they still performed fairly. The information could be explained by local factors in Hong Kong that promoted health literacy in Hong Kong people. One of the major events would be the outbreak of severe acute respiratory syndrome (SARS) in 2003. Reports have suggested Hong Kong people practiced more health-seeking behavior after the outbreak, including adoption of healthier lifestyle and good personal hygiene [33]. The government had also taken up greater effort in health promotion about infectious disease. Therefore, with the above actions, Hong Kong people may develop better health knowledge regarding antibiotics and AMR compared to global counterparts.

The study conducted a communication intervention session to raise the knowledge level regarding antibiotics and AMR in Hong Kong elderly with the effort of healthcare professionals and healthcare students. The intervention was shown to be successful as shown from the post-intervention knowledge scores for both elders and students. Past educational intervention studies on antibiotic use mostly targeted on the adult public or children at school, however, elderly are prone to having infections and need to use antimicrobials as treatment [14]. Furthermore, aging population is a global fact. Hong Kong is no exception. The decline of birth rate also led to many elders will have no children to support their care. In our current study, over 20% in the intervention group and over 40% in the control group were living alone. Therefore, it is important to education and disseminate health education to the elders in the community. They are also found to have low levels of understanding on antibiotics and so are at risk of misuse of the medications [8]. Although elders are thought to have poorer understanding and learning ability, health education is recommended to help with protective and wellness development among elders [34]. It was reflected from the study that elderly have the ability to learn and plays a role in combating AMR. Intervention programs targeting elderly should be implemented.

Concerning the mode of intervention, the study utilized both communication and multimedia in education. According to feedback from participants, around half of them agreed that the video and explanation by instructor had increased their knowledge on antibiotics and AMR. The findings echoed with previous studies, which recognized the use of multimedia in health education in elderly as an effective and efficient approach [24, 35]. Direct communications involving asking open questions, employing teach back method, and confirming responses were also shown to be effective in health education in elderly, and with active participation, the elderly could benefit from improvement in quality of life when they express themselves [36]. The study results were consistent with past study that simply one-to-one verbal education is effective in enhancing knowledge regarding antibiotic use and antibiotic resistance [37]. On top of it, the study showed that a video could be an effective tool in education when verbal explanation is provided alongside. The video would be a more efficient mode as compared with simply verbal explanation, which could be time-consuming and resourceful. However, the use of video alone takes a passive form of education, and the effectiveness varies greatly depending on the duration, content, and quality of the video [38]. The short duration of a video was likely to provide short term effects only. Use of video as a supplementary tool in health education carried beneficial effect if well designed.

Regarding the instructors of the study, students are shown to carry potential benefits in assisting primary health education. Students involving in community education is beneficial to both parties: students could learn to work effectively with communities, while the community benefits from the health services provided by students [36]. There is a great potential of health professions students to take part in health education and promotion in the community to increase awareness in public health issues. Existing research supported the community involvement of healthcare students [37]. Such practice is common in other countries including the United States. Previous study has demonstrated that a ‘train-the-trainer’ approach could enhance trainers’ knowledge on AMR [39]. Educational programs led by healthcare students in raising public awareness on proper antibiotic use was shown to be effective in increasing patient knowledge on the issue [40, 41]. The study also proved that healthcare students could help increase knowledge of antibiotics and AMR in the general public. Besides, the CHP aims to develop and implement targeted evidence-based health promotion programs for students, and states that secondary school students are key stakeholders who should take ownership to combat AMR [13]. The current study shows that training secondary school students as trainers is an effective means to raise their knowledge on AMR. Nevertheless, although there could be potential benefits of conducting student-led educational programs, concerns must be addressed before the implementation. Student-led programs were often limited to students from the medical profession since they were educated with the essential health-related knowledge. There should also be well-designed training and evaluation for the students to ensure that they are equipped with the knowledge and could be able to pass on the information. One of the method to ensure quality of student-led educational programs is to have medical professionals as preceptors [42]. Healthcare professionals at frontline could identify need for education and coordinate community intervention, while students may serve as essential resource in implementing the health education programs [37]. Therefore, there are great potentials in introducing more student-led health educational programs provided the trainers are well trained and assistance form healthcare professionals are readily available.

For the control group, there was also a statistically significant increase in knowledge even though no knowledge transfer session is provided in between. It is possible that pretests pose effects on posttests performances as the pretests could be orienting and motivational and thus provides teaching functions [43]. The increase in knowledge level may also be due to recent health promotions carried out by the CHP and WHO [13]. Previous studies also supported effectiveness of public campaign employing mass media to promote safe use of antimicrobials and increase awareness of AMR [12].

## Limitations

The study employed a pretest–posttest study approach, which focused on the knowledge about AMR attained through the knowledge transfer intervention. However, it is also important to note any changes in practices after the implementation of knowledge, for example, appropriate use of antibiotics and practice of personal hygiene. It is found from the survey on AMR conducted by the CHP that the general public holds certain wrong practice, such as requesting antibiotics from the doctors [8]. Since the actions are what matter in combating AMR, so whether such educational intervention program could motivate a change in practice is also essential. Due to limited time and difficulty in assessing such practices, evaluation of such was not included in the study.

One of the goals of educational intervention is to create outcomes that are sustainable. However, due to limited time and resources, and also to limit disturbances to participants, the study did not go through a detailed follow up to assess the sustainability of the intervention. Collection of outcome data at several different time points would be able to estimate the sustained effect of the intervention, such as identifying trends and the frequency of repeated education to maintain the knowledge in the general public [44]. Thus, future studies on intervention on AMR could focus on the above aspects.

A major challenge to implement public education would be the allocation of resources. With limited resources, the most effective and efficient health education programs are preferred. The study focused on individual education, while there could be many other different education programs. Public campaigns, pharmacist educational intervention, use of mass media are shown to be effective approaches [19, 20, 45]. Yet, more comprehensive studies could be conducted to understand the factors that contributes to a successful intervention to provide directions for policy makers [46].

Lastly, the impact of the study is limited to the public sector. AMR is a serious problem that needs to be addressed immediately. Although community perceptions hold certain effects on promoting AMR, the clinical and agriculture sectors play greater role in driving the development of AMR [6]. Current studies mainly focus on the prescribing practice, and then the community knowledge, however, limited effort has been made to educate the agriculture sector [14]. It would bring greater impact to educate farmers about AMR with the hope that such intervention would bring changes in their attitudes and practices of using antimicrobials.

## Conclusion

The study has evaluated knowledge transfer concerning AMR on Hong Kong elderly patients and students in Hong Kong. The results showed that the intervention was successful in elders, as the intervention group obtained a better score in knowledge regarding antibiotics and AMR than the control group. It is recommended to implement educational intervention on elderly and utilize multimedia in education of AMR. Still, the long-term effects and effects on behavior are uncertain.

More could be done to evaluate different education interventions concerning AMR. Better study design would be one of the aspects, including more rigorous designs like randomized controlled trials, and more comprehensive outcome measures. Well-designed studies could provide more directions in allocating resources in health promotion in the community.

## Data Availability

The datasets used and/or analyzed during the current study are available from the corresponding author on reasonable request.

## Abbreviations

AMR: Antimicrobial resistance
CHAMPION: Community Health and Medication-safety Promotion Inter-schools Outreach Network
CHP: Centre for Health Protection
CUHK: Chinese University of Hong Kong
SARS: Severe acute respiratory syndrome
WHO: World Health Organization
